# Supplementary material for: Analysis of Potential Biomarkers in Frontal Temporal Dementia: A Bioinformatics Approach
Source: Int J Mol Sci. 2023 Oct 5;24(19):14910. doi: 10.3390/ijms241914910 (PMC10573524; doi:10.3390/ijms241914910)
Supplement: Supplementary file 1 [file ijms-24-14910-s001.zip › Supplementary_Figures.pptx]

## Slide 1
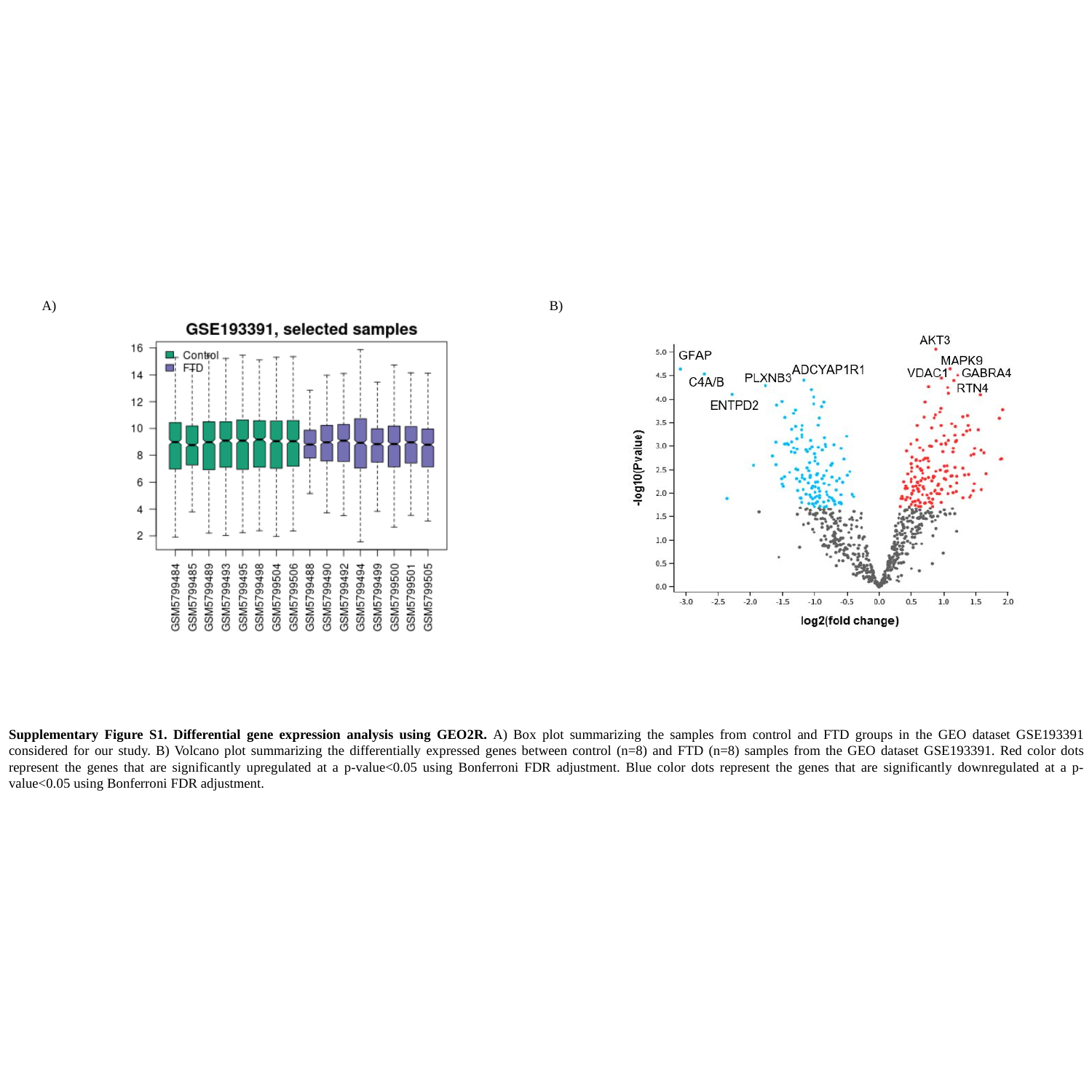

A)
B)
Supplementary Figure S1. Differential gene expression analysis using GEO2R. A) Box plot summarizing the samples from control and FTD groups in the GEO dataset GSE193391 considered for our study. B) Volcano plot summarizing the differentially expressed genes between control (n=8) and FTD (n=8) samples from the GEO dataset GSE193391. Red color dots represent the genes that are significantly upregulated at a p-value<0.05 using Bonferroni FDR adjustment. Blue color dots represent the genes that are significantly downregulated at a p-value<0.05 using Bonferroni FDR adjustment.

## Slide 2
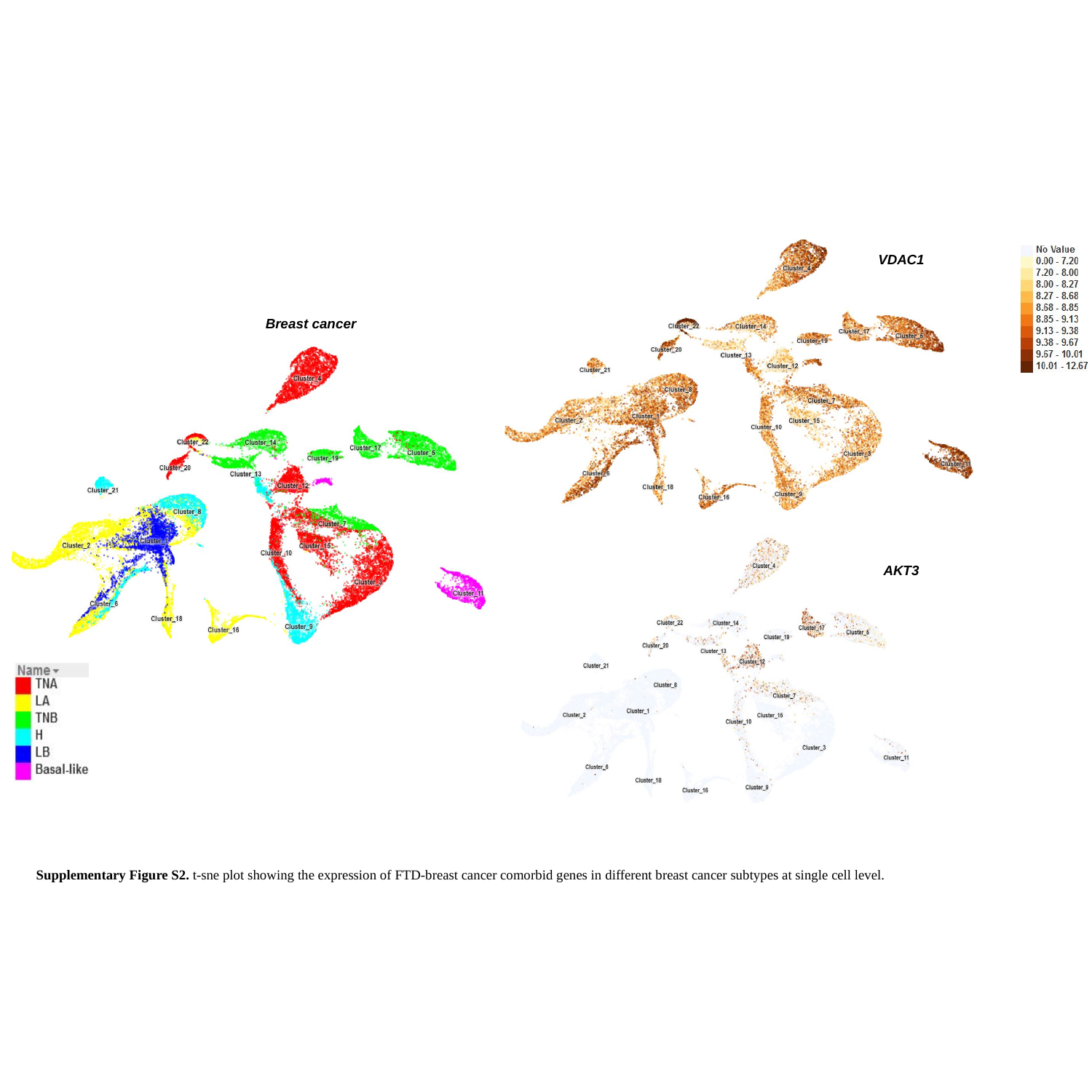

VDAC1
Breast cancer
AKT3
Supplementary Figure S2. t-sne plot showing the expression of FTD-breast cancer comorbid genes in different breast cancer subtypes at single cell level.

## Slide 3
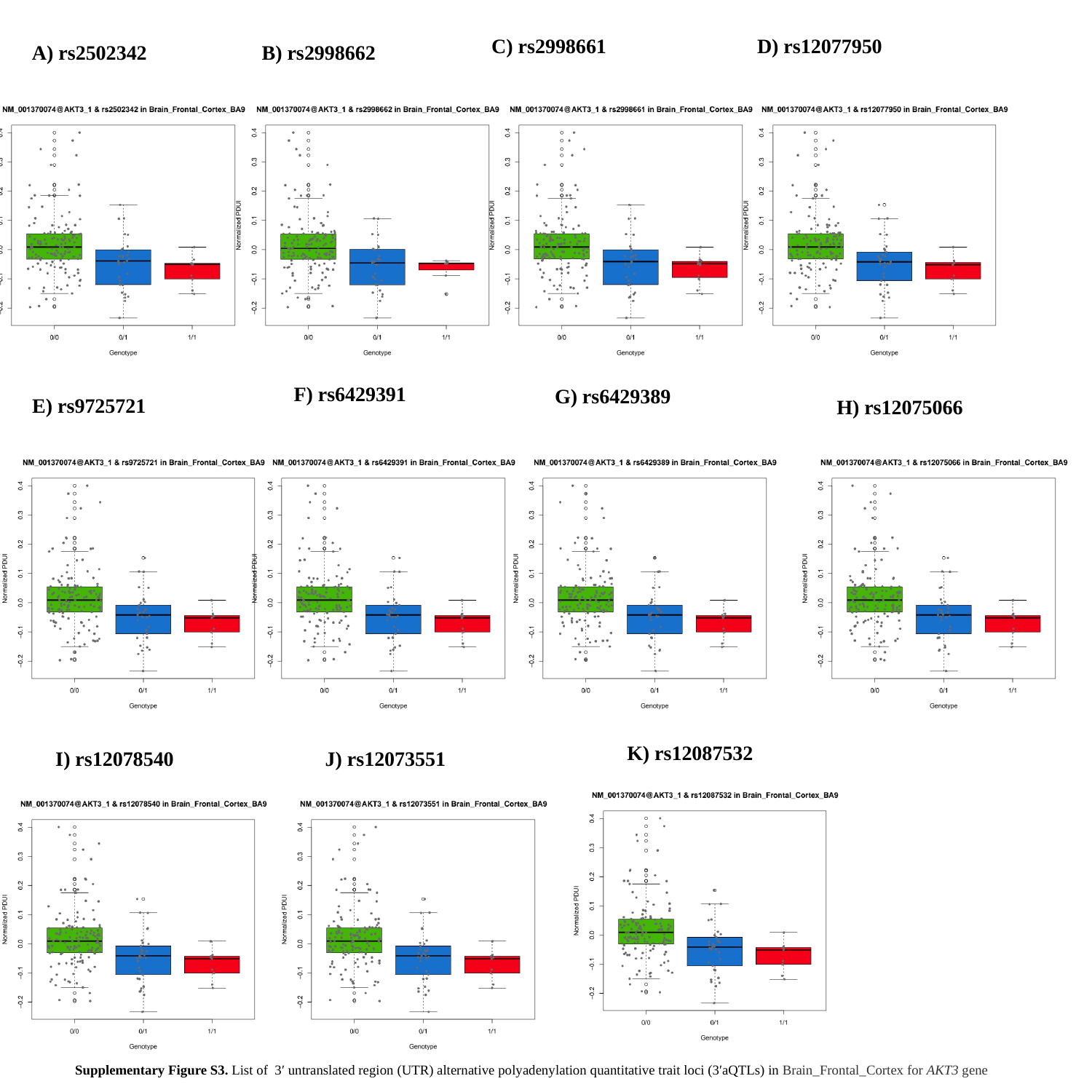

C) rs2998661
D) rs12077950
A) rs2502342
B) rs2998662
F) rs6429391
G) rs6429389
E) rs9725721
H) rs12075066
K) rs12087532
I) rs12078540
J) rs12073551
Supplementary Figure S3. List of  3′ untranslated region (UTR) alternative polyadenylation quantitative trait loci (3′aQTLs) in Brain_Frontal_Cortex for AKT3 gene

## Slide 4
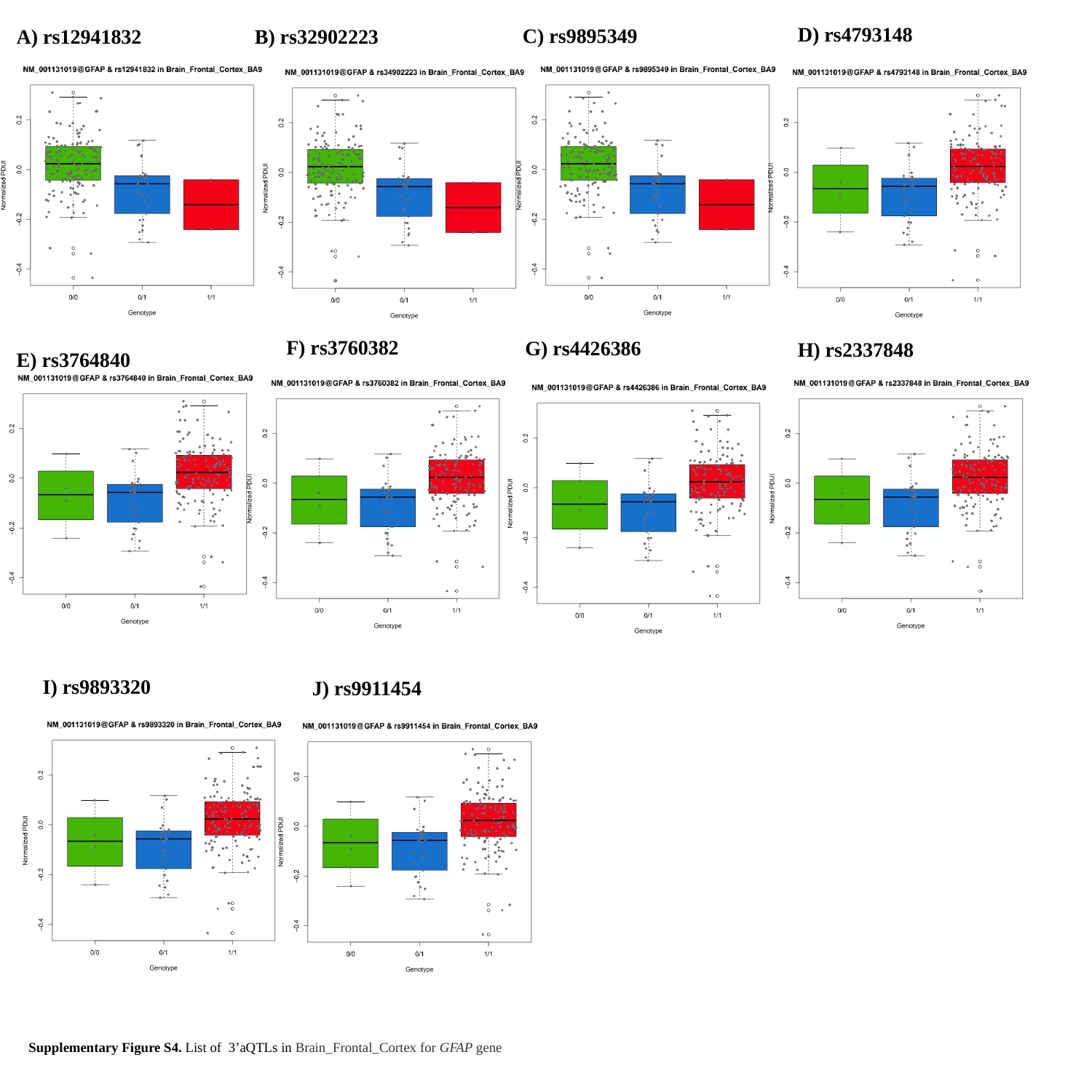

D) rs4793148
C) rs9895349
A) rs12941832
B) rs32902223
F) rs3760382
G) rs4426386
H) rs2337848
E) rs3764840
I) rs9893320
J) rs9911454
Supplementary Figure S4. List of  3’aQTLs in Brain_Frontal_Cortex for GFAP gene

## Slide 5
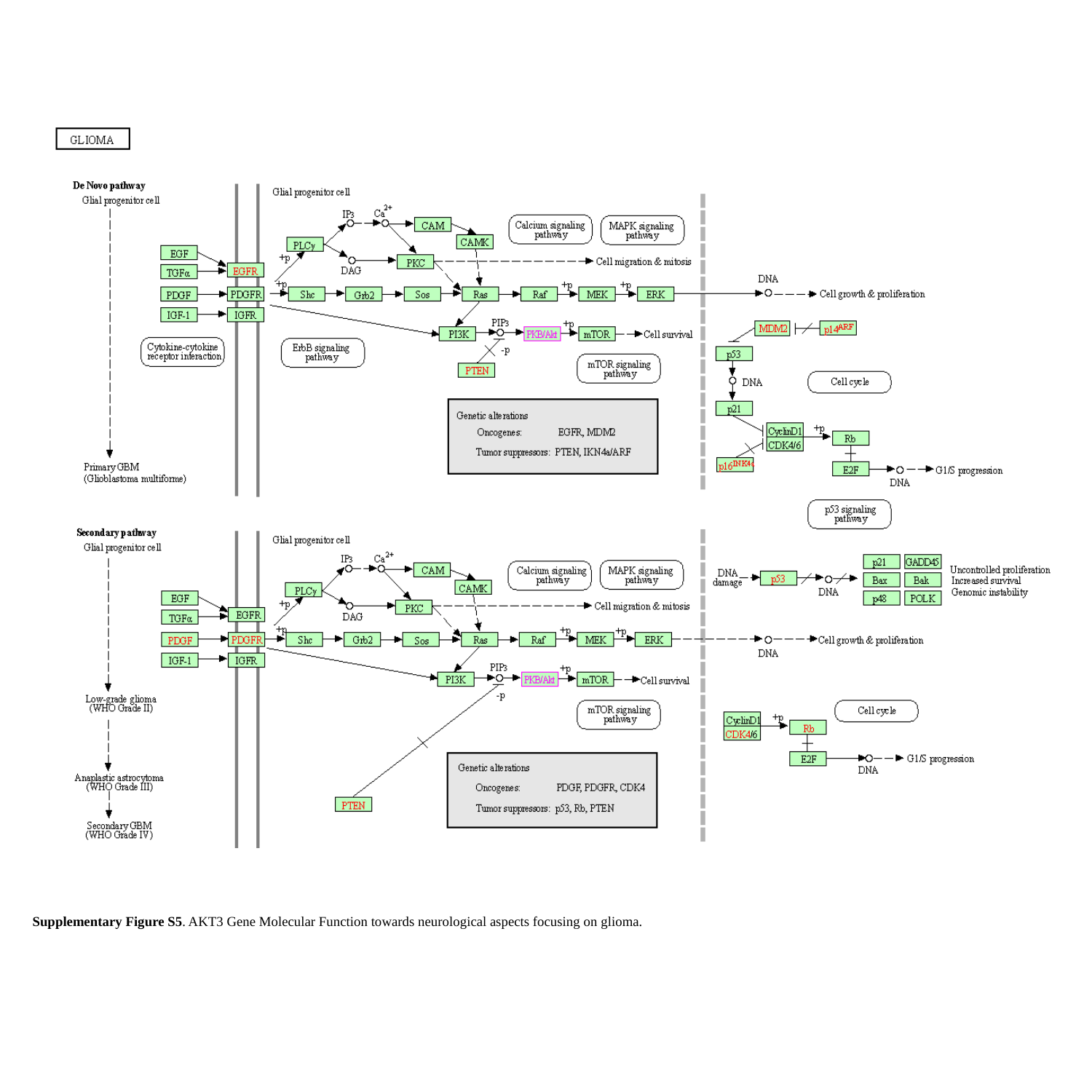

Supplementary Figure S5. AKT3 Gene Molecular Function towards neurological aspects focusing on glioma.

## Slide 6
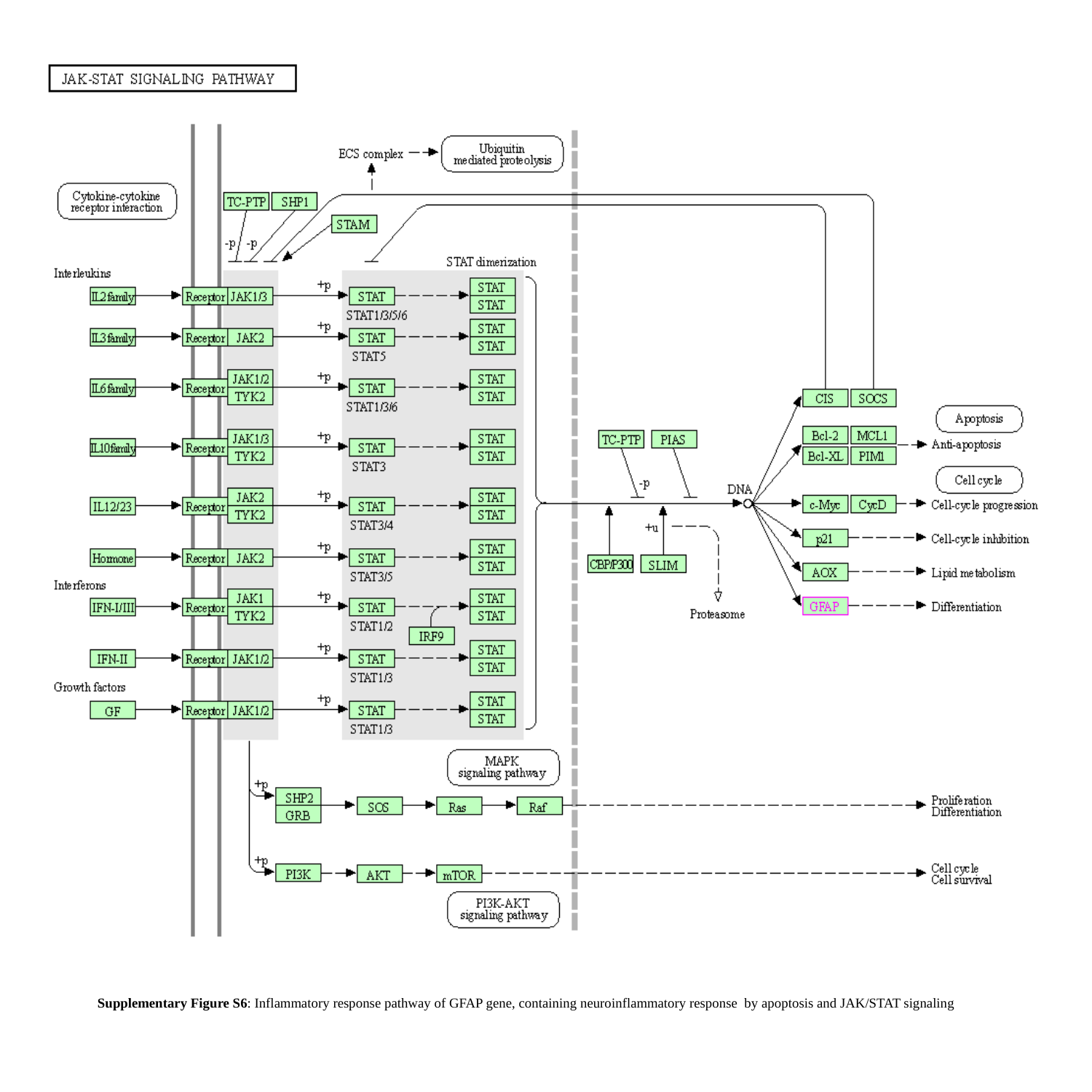

Supplementary Figure S6: Inflammatory response pathway of GFAP gene, containing neuroinflammatory response  by apoptosis and JAK/STAT signaling

## Slide 7
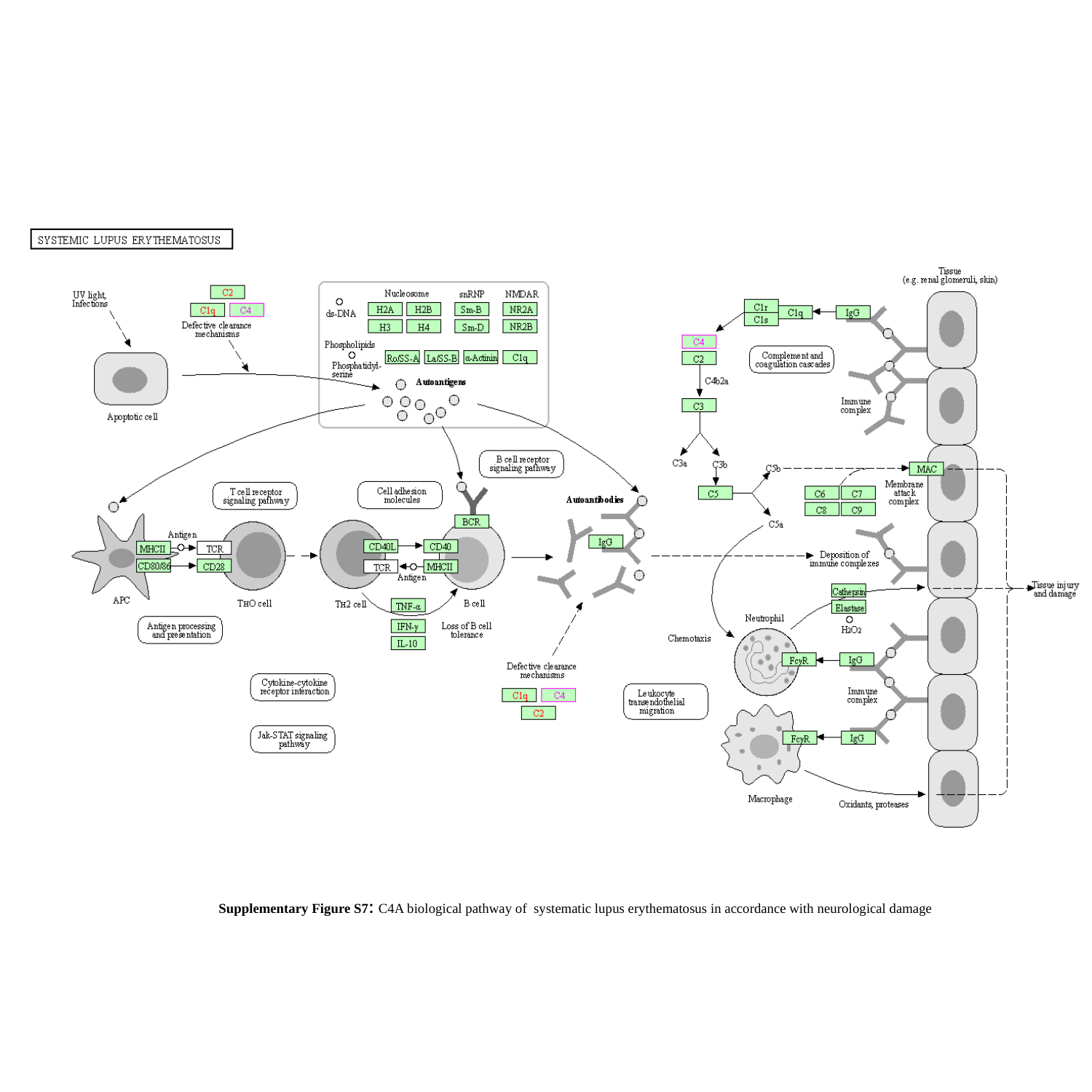

Supplementary Figure S7: C4A biological pathway of  systematic lupus erythematosus in accordance with neurological damage

## Slide 8
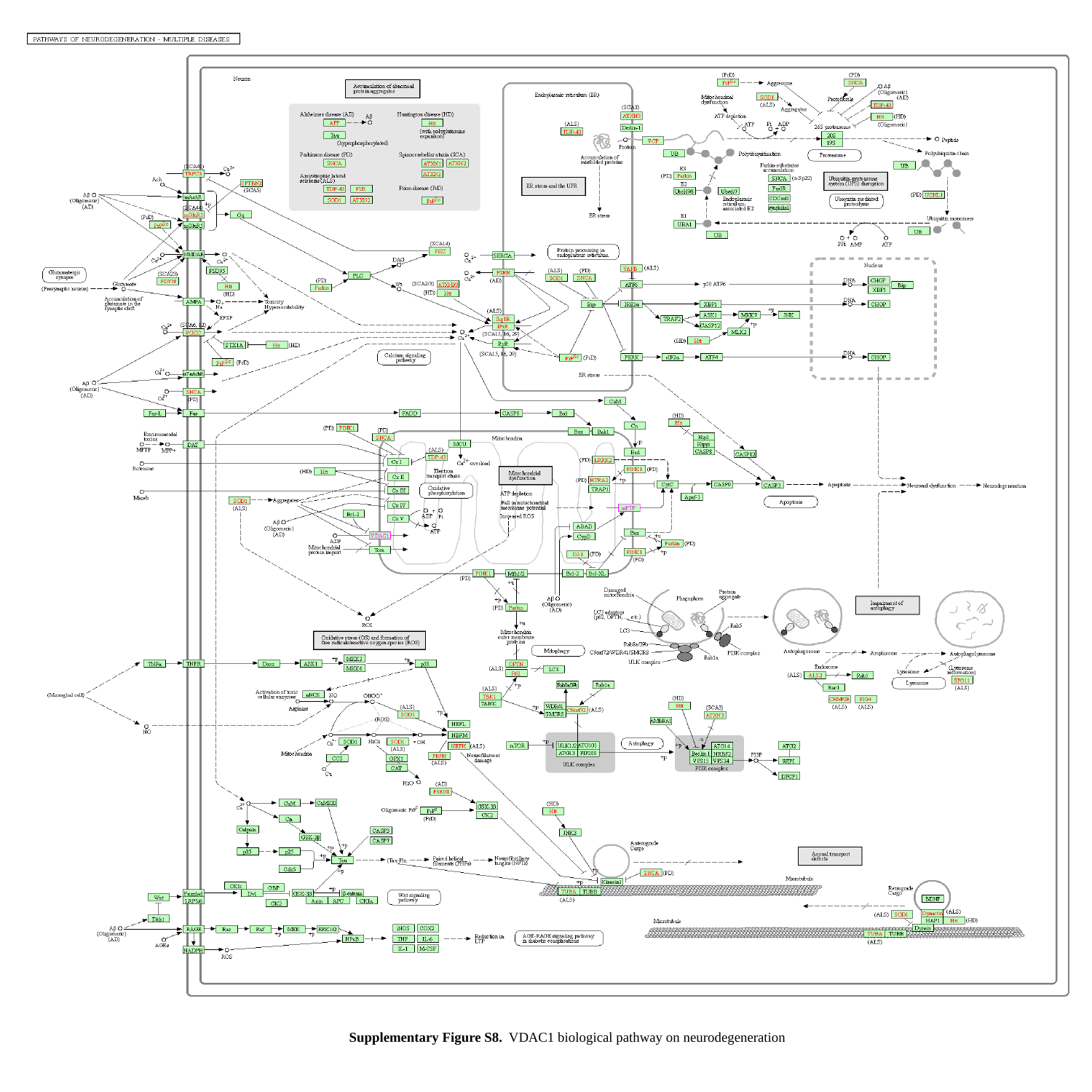

Supplementary Figure S8.  VDAC1 biological pathway on neurodegeneration

## Slide 9
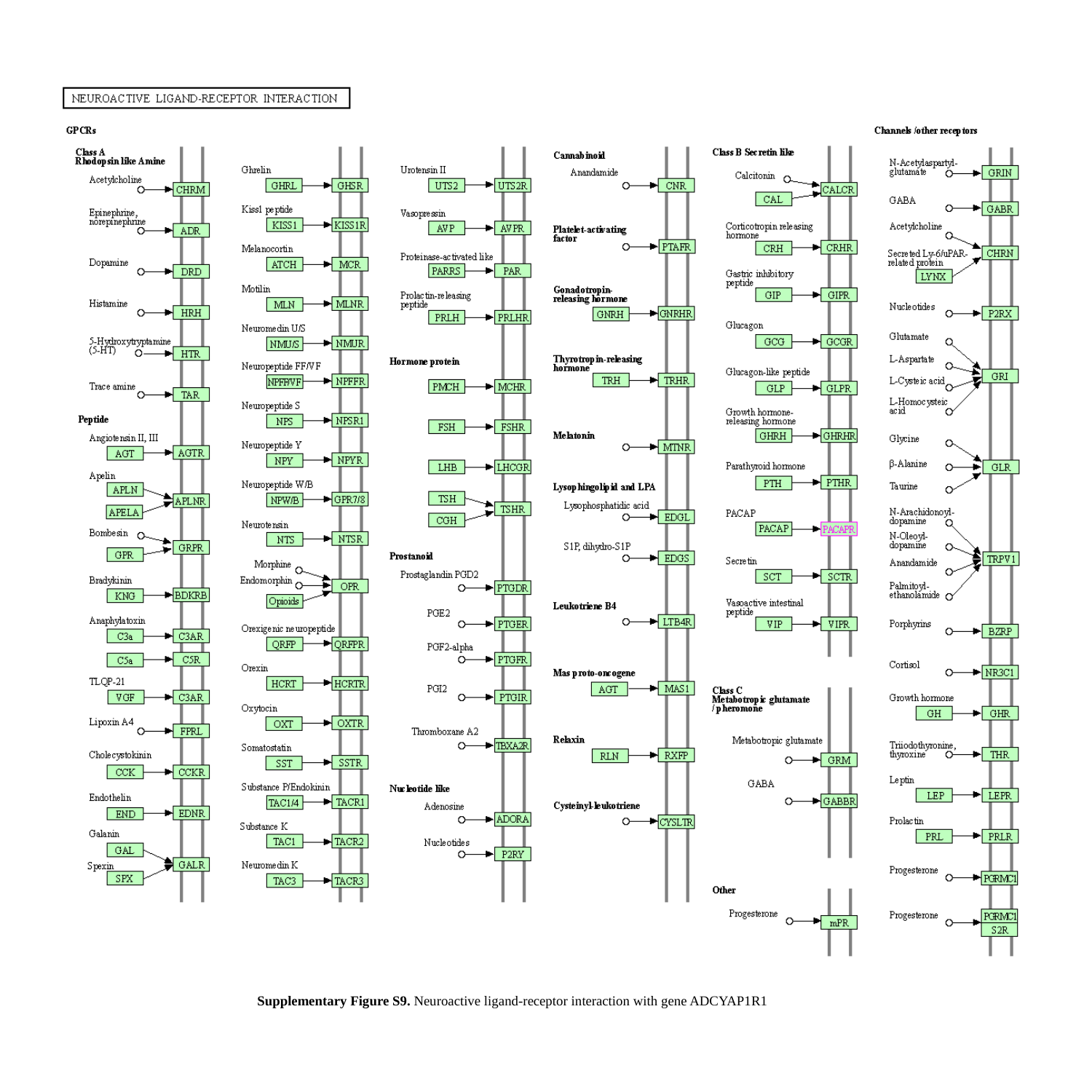

Supplementary Figure S9. Neuroactive ligand-receptor interaction with gene ADCYAP1R1
