# Supplementary material for: Analysis of Potential Biomarkers in Frontal Temporal Dementia: A Bioinformatics Approach
Source: Int J Mol Sci. 2023 Oct 5;24(19):14910. doi: 10.3390/ijms241914910 (PMC10573524; doi:10.3390/ijms241914910)
Supplement: Supplementary file 1 [file ijms-24-14910-s001.zip › Supplementary_Tables.pdf]

Supplementary Table S1. Samples from the GEO dataset GSE193391 that are considered for our study.

**Control Samples**				
**Accession**	**Age**	**Sex**	**Race**	**APOE status**
GSM5799484	52	Female	White	E3/4
GSM5799495	59	Male	Black	E2/3
GSM5799498	57	Male	White	E3/3
GSM5799485	78	Female	White	E3/3
GSM5799504	56	Male	White	NA
GSM5799489	70	Male	Black	E3/3
GSM5799493	75	Female	White	E3/3
GSM5799506	74	Female	White	E3/3
**FTD Samples**				
**Accession**	**Age**	**Sex**	**Race**	**APOE status**
GSM5799499	81	Male	White	E3/3
GSM5799500	60	Female	White	E3/4
GSM5799501	64	Male	White	E3/4
GSM5799488	71	Male	White	NA
GSM5799505	56	Female	White	NA
GSM5799490	65	Female	White	E2/3
GSM5799492	65	Female	White	NA
GSM5799494	63	Male	White	E3/3

Supplementary Table S2. List of genes that are differentially expressed between control and FTD samples from African American and white ethnic background

ID	log2(fold change)	Log10(Pvalue)
RAB3A - mRNA	1.915144402	3.77847022
NEFL - mRNA	1.897532002	2.733218677
KCNV1 - mRNA	1.885089293	2.719638972
BDNF - mRNA	1.866737397	3.591163588
YWHAH - mRNA	1.6608987	2.405732385
GABRG2 - mRNA	1.628626591	2.853410941
SH3GL2 - mRNA	1.583076732	2.06976279
SYN2 - mRNA	1.57975656	2.906948704
RIMS1 - mRNA	1.568248876	4.093696854
PAK1 - mRNA	1.536618015	3.345044705
LRRC7 - mRNA	1.483228062	2.949637769
OLFM3 - mRNA	1.478048842	2.196272335
NSF - mRNA	1.470471918	2.8267357
SYT4 - mRNA	1.463409341	2.047512958
RTN1 - mRNA	1.424424538	2.802983479
SNAP25 - mRNA	1.421016388	1.947949038
PTK2B - mRNA	1.410402443	3.341846011
NAP1L2 - mRNA	1.403716046	1.904585667
EGR1 - mRNA	1.384945572	2.389110077
CAMK2A - mRNA	1.378496175	3.43386502
NUAK1 - mRNA	1.369764371	3.644423765
GNG2 - mRNA	1.357187146	3.320099298
GOT1 - mRNA	1.325792453	2.378155293
INPP5F - mRNA	1.305575319	3.243018577
WASF1 - mRNA	1.301920962	1.892321479
PPP3R1 - mRNA	1.30075068	2.529676746
STXBP1 - mRNA	1.300095155	3.621653783

GABRA4 - mRNA	1.219070941	4.509554079
DNM1 - mRNA	1.201255269	2.352457955
GLRB - mRNA	1.200900947	2.215614688
ATP6V1A - mRNA	1.196505702	3.115400512
ATP1B1 - mRNA	1.193819927	2.031703794
GLRA3 - mRNA	1.183966166	3.092616856
KCNQ3 - mRNA	1.179882463	3.412678642
CHN1 - mRNA	1.174702891	2.544797267
CAMK4 - mRNA	1.173708395	3.232712429
RTN4 - mRNA	1.15831902	4.394279735
ATP8A2 - mRNA	1.14966702	2.950537222
SCN8A - mRNA	1.146906285	2.008116859
GABRB3 - mRNA	1.145156702	2.87116907
SLC9A6 - mRNA	1.131125193	2.361239462
RAB6B - mRNA	1.126458729	3.474648238
ADAM22 - mRNA	1.116226691	2.356014975
OPCML - mRNA	1.107954773	2.135919814
MAPK9 - mRNA	1.103027034	4.643847565
GAS7 - mRNA	1.076166641	4.123247843
GABRD - mRNA	1.076020515	3.117773673
GNG3 - mRNA	1.0698848	1.987961021
ELOC - mRNA	1.064341266	4.247940241
DLGAP1 - mRNA	1.058353261	2.472027972
SCOC - mRNA	1.055877723	2.578757727
CHRM3 - mRNA	1.042085502	2.447089994
GRIA3 - mRNA	1.035749257	3.037924991
GPRASP1 - mRNA	1.033593913	2.287202604
EPB41L3 - mRNA	1.031961645	2.467957958
VDAC3 - mRNA	1.030329792	3.060049353
CD200 - mRNA	1.025489227	2.450437883

TSPAN7 - mRNA	1.00912407	2.571620596
ATP6V1B2 - mRNA	1.007374069	2.305591583
GRM7 - mRNA	1.003906517	3.22158249
NPTN - mRNA	0.986291875	2.878055769
SYN1 - mRNA	0.96976014	2.070087775
ELMO1 - mRNA	0.966630928	2.077737383
VDAC1 - mRNA	0.965119399	4.443380835
RASGRF1 - mRNA	0.962469575	3.04267522
DCLK1 - mRNA	0.962464282	1.797943437
SHC3 - mRNA	0.961235177	3.433607734
ACSL4 - mRNA	0.959394811	3.80475451
ATP6V1C1 - mRNA	0.948824655	2.490877896
DNAJC5 - mRNA	0.943238687	3.72288062
ATP1A1 - mRNA	0.935891012	1.985968873
THY1 - mRNA	0.923503371	2.192981444
CUX2 - mRNA	0.913827343	2.155251033
MAL2 - mRNA	0.902747239	1.990336016
CDK5R1 - mRNA	0.888240135	3.108818381
AKT3 - mRNA	0.882159071	5.067588548
NDUFB5 - mRNA	0.88202676	2.462037282
CCNA1 - mRNA	0.874553085	3.660615382
ERLEC1 - mRNA	0.870932918	2.056460252
GPM6A - mRNA	0.859561301	2.347365339
NCAN - mRNA	0.859469661	2.038926235
TNFRSF11A - mRNA	0.853762528	3.599233881
PRKACB - mRNA	0.838274822	2.353137015
CD47 - mRNA	0.838044401	2.324197232

CHRNB2 - mRNA	0.82749162	1.863484769
TUSC3 - mRNA	0.826263872	1.713636951
GLS - mRNA	0.814156846	2.259348658
PPP2CA - mRNA	0.812230706	3.383232914
GRIN2A - mRNA	0.810991331	1.783510405
MAP2K4 - mRNA	0.809194743	2.996969002
RORB - mRNA	0.802570777	1.992757562
ATP6V0D1 - mRNA	0.783279941	3.003200326
ZNF365 - mRNA	0.782645652	1.805601788
YWHAZ - mRNA	0.767980763	1.884538749
LGMN - mRNA	0.767724646	2.675968969
UBE2N - mRNA	0.766382926	4.269228862
NLK - mRNA	0.765657757	2.327488828
CYFIP2 - mRNA	0.763266633	2.544689517
TANC2 - mRNA	0.751802158	2.74408857
PRKCZ - mRNA	0.74386205	1.957067863
ATP6V1E1 - mRNA	0.71899494	1.787761678
ATP5MC3 - mRNA	0.716880176	2.742671217
ARPC5L - mRNA	0.715565935	2.296888637
HK1 - mRNA	0.714724418	3.933186341
FGF13 - mRNA	0.707558202	1.780608037
NRCAM - mRNA	0.699394099	3.613846441
DNAJA2 - mRNA	0.696784447	2.085295738
SEC61A2 - mRNA	0.6852858	2.711823267
BASP1 - mRNA	0.68366809	2.132921611
HSP90AB1 - mRNA	0.683387239	2.701011871
DLAT - mRNA	0.682127404	2.906699187

OPA1 - mRNA	0.677233346	2.118651084
ATP6V1D - mRNA	0.67578652	2.417188509
CALM1 - mRNA	0.67231428	1.966211372
DIAPH1 - mRNA	0.671718791	2.312973248
CACNA2D1 - mRNA	0.664952207	2.485190098
MAD2L1 - mRNA	0.651720161	2.482135437
NKIRAS1 - mRNA	0.643702183	2.262960325
GNAI1 - mRNA	0.640217588	1.714972328
MAPK8 - mRNA	0.634886871	2.518950744
GRIA2 - mRNA	0.633101085	1.795734936
CLTC - mRNA	0.631539492	2.682689277
PSMA5 - mRNA	0.627739528	2.96244203
TRIM3 - mRNA	0.625796777	2.160900959
SYNGR1 - mRNA	0.613098286	2.033077213
ATP6V1H - mRNA	0.61280585	1.715251035
NCKAP1 - mRNA	0.606602325	1.844293695
PGK1 - mRNA	0.60119424	2.077691906
TXN - mRNA	0.596388953	2.223449833
ATG5 - mRNA	0.59631649	2.087479756
B4GALT5 - mRNA	0.592375921	2.234431387
STAMBPL1 - mRNA	0.591910365	2.562838417
ZPR1 - mRNA	0.589918468	3.434021774
ACLY - mRNA	0.586144442	3.12739573
PSMC6 - mRNA	0.575497493	2.714056811
MAPK1 - mRNA	0.572836084	2.687324183
TNFRSF21 - mRNA	0.572146782	2.144145178
GABARAPL1 - mRNA	0.564215775	1.738782735

APP - mRNA	0.560003051	1.821531602
MEAF6 - mRNA	0.55572239	2.33573698
REEP5 - mRNA	0.534216313	2.051347495
RLIM - mRNA	0.528364097	2.131842654
SHANK2 - mRNA	0.525302598	1.808518577
ACSL3 - mRNA	0.512476292	2.765626134
YWHAE - mRNA	0.510352923	1.997561794
CSNK2A1/3 - mRNA	0.508070203	2.536197367
DYNC1LI1 - mRNA	0.507608722	1.979187294
GSK3B - mRNA	0.502941772	2.4575882
ABI2 - mRNA	0.502550718	2.620434724
AP3M2 - mRNA	0.498920213	1.754309728
ATP2A2 - mRNA	0.498462148	2.294913287
FIG4 - mRNA	0.496092259	2.24362366
BRAF - mRNA	0.492302539	3.045037412
ANAPC1 - mRNA	0.491267133	2.618024011
KRAS - mRNA	0.48435897	1.881810293
DNM3 - mRNA	0.465614224	1.704831045
CUL1 - mRNA	0.44181277	2.400804532
ULK1 - mRNA	0.433505093	1.904689803
SERINC3 - mRNA	0.431901038	2.096997932
GNB1 - mRNA	0.423548346	2.892864251
ATRN - mRNA	0.412060634	1.850877956
PRKCI - mRNA	0.411938315	2.144298639
RAD23B - mRNA	0.404755516	2.092129219
KPNA1 - mRNA	0.381365508	2.241218693
ABCE1 - mRNA	0.363557028	1.878343697
PDHA1 - mRNA	0.35717273	1.931822902
ATP9A - mRNA	0.345132246	1.876206705

PSMD14 - mRNA	0.329644117	1.713425726
CTSD - mRNA	-0.393157053	1.920089355
JAK1 - mRNA	-0.416864604	1.974333465
RECK - mRNA	-0.45556215	2.453338079
TICAM1 - mRNA	-0.496543176	2.373288141
HSD17B4 - mRNA	-0.505298988	3.206893239
STAT2 - mRNA	-0.546190807	2.726190105
GAS6 - mRNA	-0.564993165	2.231567017
AKT1 - mRNA	-0.586231835	1.770457928
PKD2 - mRNA	-0.587444597	2.949793233
DLX2 - mRNA	-0.58971835	1.964722442
BHLHE41 - mRNA	-0.594814879	1.796635386
SLC46A1 - mRNA	-0.615250765	2.307265974
NINJ2 - mRNA	-0.623288297	1.79239465
OLIG2 - mRNA	-0.660888561	2.258969711
TJP1 - mRNA	-0.670529882	2.062996634
SCD5 - mRNA	-0.687825965	1.758334233
DLX1 - mRNA	-0.697677515	1.820857037
SCP2 - mRNA	-0.702742456	3.028781995
ITGAM - mRNA	-0.712150444	1.843638005
CNP - mRNA	-0.728876524	2.624542907
ADGRA2 - mRNA	-0.732452577	2.336367627
PTGIS - mRNA	-0.734615836	1.884051201
ENTPD1 - mRNA	-0.746925918	2.588440584
ITM2A - mRNA	-0.771108243	1.912806701
EGF - mRNA	-0.775219311	2.172442703
ERMN - mRNA	-0.784402119	1.833821456
ITGAX - mRNA	-0.806770926	1.963235045
AHCYL1 - mRNA	-0.814555336	1.719177829
CPNE2 - mRNA	-0.816474996	2.477271213

CYSLTR1 - mRNA	-0.818440428	2.024348128
HSD11B1 - mRNA	-0.829698574	2.98963908
SALL1 - mRNA	-0.836885748	1.702787451
LAMP2 - mRNA	-0.838638926	3.034281228
TIMP2 - mRNA	-0.852727158	3.174280616
CSPG4 - mRNA	-0.855282168	1.699097591
ITGA7 - mRNA	-0.858253307	2.203417138
GRN - mRNA	-0.860959026	3.931396962
P2RY11 - mRNA	-0.861122587	2.363630703
IL21R - mRNA	-0.863811934	1.805678387
SOX2 - mRNA	-0.884088718	2.912064408
CCR5 - mRNA	-0.887486059	2.303424229
GJB1 - mRNA	-0.89409743	3.840177354
CEACAM3 - mRNA	-0.917447635	1.718815784
MS4A1 - mRNA	-0.922784681	1.894304516
KLRK1 - mRNA	-0.923118215	2.03734365
HLA-A - mRNA	-0.924505983	1.950258982
MAFB - mRNA	-0.928106095	3.596527029
LSR - mRNA	-0.937991062	2.099391279
MERTK - mRNA	-0.942230697	2.973753083
CNTF - mRNA	-0.945181184	3.440543396
PLP1 - mRNA	-0.950394766	2.616822875
PTGER4 - mRNA	-0.953627861	2.238905125
TMEM119 - mRNA	-0.955240123	2.408504381
NOSTRIN - mRNA	-0.95620864	1.812194123
CD9 - mRNA	-0.965362371	1.928068069
SELENOS - mRNA	-0.966025536	2.381926915
TFEC - mRNA	-0.979529846	1.783661122
ALDH1L1 - mRNA	-0.988282388	2.232382152

OLIG1 - mRNA	-0.988747174	2.127124767
IL10RA - mRNA	-0.989385237	2.76211199
SOX10 - mRNA	-0.994836926	2.677931345
NLRP1 - mRNA	-0.995131928	2.01611036
CTSS - mRNA	-0.996525135	1.896037781
FA2H - mRNA	-0.999281332	2.836512415
KCND1 - mRNA	-1.003038114	2.687292711
SLC2A5 - mRNA	-1.004509425	1.713068445
SLCO2B1 - mRNA	-1.008026648	2.834218529
PMP22 - mRNA	-1.014416151	4.048081157
ERBB3 - mRNA	-1.015788801	3.895007325
PRF1 - mRNA	-1.025002674	1.759210446
CSF1 - mRNA	-1.032192991	2.13951733
TLR7 - mRNA	-1.035948571	1.770316797
MYRF - mRNA	-1.036655394	1.869629617
CD33 - mRNA	-1.0445459	2.984907092
C1S - mRNA	-1.047837809	2.101137736
PLXDC2 - mRNA	-1.053157797	4.198921334
MAG - mRNA	-1.062027221	1.908761951
UGT8 - mRNA	-1.065450174	3.115791754
ALDH1A1 - mRNA	-1.070393671	2.369309764
PTPRC - mRNA	-1.073119834	2.329953915
TGM1 - mRNA	-1.075140124	2.938760734
KLRB1 - mRNA	-1.077875323	1.96157755
SPI1 - mRNA	-1.081145312	2.640229366
KIR2DL3 - mRNA	-1.090713511	1.774319322
P2RY14 - mRNA	-1.092290684	2.475477055
HDC - mRNA	-1.099492654	2.216447345
TSPO - mRNA	-1.113641609	2.881174716
TIE1 - mRNA	-1.118034156	2.535422157

CD3D - mRNA	-1.13214563	1.97727806
TGFB1 - mRNA	-1.133335462	2.91861679
CLDN5 - mRNA	-1.154996705	2.295071942
ADCYAP1R1 - mRNA	-1.166703069	4.400938125
CLEC7A - mRNA	-1.178811097	2.071823739
TBX21 - mRNA	-1.194747791	2.375802367
EGFR - mRNA	-1.195300507	3.433184742
FAM30A - mRNA	-1.196299873	2.325579871
KCTD12 - mRNA	-1.198689902	3.187341701
LILRB4 - mRNA	-1.199190275	3.242314051
C3AR1 - mRNA	-1.202783824	1.79753248
AQP4 - mRNA	-1.205206913	3.344658196
ADORA3 - mRNA	-1.213055577	1.883250749
MS4A4A - mRNA	-1.227267878	1.888386446
CD109 - mRNA	-1.235071695	2.247720553
VIM - mRNA	-1.265467861	2.051753652
SELPLG - mRNA	-1.266346765	2.496666015
TLR3 - mRNA	-1.274374674	2.934684593
P2RY1 - mRNA	-1.277152616	3.150082053
LAIR1 - mRNA	-1.281506596	2.304075187
CSF1R - mRNA	-1.289868506	2.513658092
RHOB - mRNA	-1.294972863	3.414023611
SOX9 - mRNA	-1.297539906	3.769367195
RFX4 - mRNA	-1.307713927	2.858941921
AGMO - mRNA	-1.324806147	2.959027559
FCGRT - mRNA	-1.325799855	3.695450178
LRRC25 - mRNA	-1.355495124	3.355357204
PLEKHB1 - mRNA	-1.356334235	3.087056281
TLR4 - mRNA	-1.363957215	2.873748979

ABCA1 - mRNA	-1.390588748	2.437425864
TLR2 - mRNA	-1.414107956	3.034313079
SERPING1 - mRNA	-1.432977066	3.05399254
GDPD2 - mRNA	-1.463402045	3.608418821
IGFBP5 - mRNA	-1.466368999	3.044012993
S100A10 - mRNA	-1.469458217	2.343770131
SLC11A1 - mRNA	-1.487931058	2.137304111
SIGLEC5 - mRNA	-1.503273686	2.193316068
CSF3R - mRNA	-1.505708199	3.943722943
CFI - mRNA	-1.509878234	2.303204162
GSN - mRNA	-1.595054098	3.870541338
NWD1 - mRNA	-1.603964624	2.599739074
SPARC - mRNA	-1.605325113	3.081767932
IL18 - mRNA	-1.655704436	2.78974419
PLXNB3 - mRNA	-1.766036402	4.291146844
CD163 - mRNA	-1.949612756	2.588219644
ENTPD2 - mRNA	-2.279332349	4.101361828
CD44 - mRNA	-2.364184755	1.876769291
C4A/B - mRNA	-2.716240375	4.539406814
GFAP - mRNA	-3.08847536	4.638839401

Supplementary Table S3. DisGeNET analysis of genes *GFAP* and *RTN4* involved in COVID-19 and FTD

Gene	DPI	DSI	pLi
GFAP	0.885	0.421	1.46E-06
RTN4	0.692	0.621	0.45318

Supplementary Table S4. List of approved and immunotherapeutic drugs interactions for the FTD-breast cancer comorbid genes using the drug gene interaction database (DGIdb) https://www.dgidb.org/search_interactions

AKT3	*VDAC1*	*ADCYAP1R1*	*GFAP*
**Drug Name (Interaction Score)**	**Drug Name (Interaction Score)**	**Drug Name (Interaction Score)**	**Drug Name (Interaction Score)**
XL-418 (2.25)	OLESOXIME (15.46)	NALOXONE (5.62)	SORBITOL (1.65)
IPATASERTIB (1.61)		INSULIN (1.21)	PERMETHRIN (1.24)
AFURESERTIB (1.41)			FADROZOLE (0.99)
TRICIRIBINE (1.12)			DEMECOLCINE (0.82)
MK-2201 (0.94)			OPRELVEKIN (0.82)
MIRANSERTIB (0.94)			DRONABINOL (0.62)
AZD-5363 (0.94)			AMPHETAMINE (0.57)
SR-13668 (0.94)			PIOGLITAZONE (0.33)
ARCHEXIN (0.94)			CAPSAICIN (0.33)
MSC-2363318A (0.94)			NIMODIPINE (0.33)
TRICIRIBINE PHOSPHATE (0.94)			PROPRANOLOL (0.31)
UPROSERTIB (0.94)			PRASTERONE (0.27)
MK-2206 (0.83)			METHIMAZOLE (0.27)
CAPIVASERTIB (0.62)			NIFEDIPINE (0.21)
LY-2780301 (0.56)			MELATONIN (0.19)
PERIFOSINE (0.56)			CAFFEINE (0.18)
GSK-690693 (0.33)			STAUROSPORINE (0.18)
VEMURAFENIB (0.19)			ISOPROTERENOL (0.18)
OMIPALISIB (0.15)			COLCHICINE (0.16)
FASUDIL (0.09)			PENTOXIFYLLINE (0.15)
EVEROLIMUS (0.07)			DISULFIRAM (0.13)
GSK-269962A (0.06)			LITHIUM (0.11)
			GENISTEIN (0.1)
			PROGESTERONE (0.07)
			ASPIRIN (0.07)

Supplementary Table S5: List of RNA binding proteins interacting with the FTD-breast cancer comorbid genes *AKT3, VDAC1, ADCYAP1R1, GFAP, C4A*

RBP	gene ID	Gene Name	Tissue	Disease Num	Diseases	cosmicNum	Sample Num	mutType Num	clipExp Num	Clip Num
EIF4A3	ENSG00000117020	*AKT3*	breast	2	ductal carcinoma, carcinoma	3	3	1	1	2
FBL	ENSG00000117020	*AKT3*	breast	3	ductal carcinoma,ductolobular carcinoma, carcinoma	5	5	1	2	4
FMR1	ENSG00000117020	*AKT3*	breast	6	ductal carcinoma,ductolobular carcinoma-PR-positive carcinoma, lobular carcinoma, basal (triple-negative) carcinoma, carcinoma	12	12	3	4	12
IGF2BP1	ENSG00000117020	*AKT3*	breast	5	duct lobular carcinoma-PR-positive carcinoma, lobular carcinoma, basal (triple-negative) carcinoma, carcinoma	7	7	3	1	6
IGF2BP2	ENSG00000117020	*AKT3*	breast	6	ductal carcinoma,ductolobular carcinoma, ER-PR-positive carcinoma, lobular carcinoma, basal	12	12	3	5	18

					(triple-negative) carcinoma, carcinoma					
IGF2BP3	ENSG0000117020	*AKT3*	breast	5	ductal carcinoma,ductolobular carcinoma, ER-PR-positive carcinoma, basal (triple-negative) carcinoma, carcinoma	7	7	3	1	4
MOV10	ENSG0000117020	*AKT3*	breast	5	ductal carcinoma,ductolobular carcinoma, ER-PR-positive carcinoma, basal (triple-negative) carcinoma, carcinoma	7	7	2	4	10
NOP56	ENSG0000117020	*AKT3*	breast	1	carcinoma	1	1	1	1	1
NOP58	ENSG0000117020	*AKT3*	breast	3	ductolobular carcinoma, basal (triple-negative) carcinoma, carcinoma	4	4	3	2	6
RBM47	ENSG0000117020	*AKT3*	breast	5	ductal carcinoma,ductolobular carcinoma, lobular carcinoma, basal (triple-negative) carcinoma, carcinoma	6	6	2	1	4
SRSF1	ENSG0000	*AKT3*	breast	2	ductal carcinoma, carcinoma	2	2	1	1	1

	17020									
EIF4A3	ENSG00000213585	*VDAC1*	breast	2	metaplastic carcinoma, carcinoma	3	3	2	2	4
IGF2BP2	ENSG00000213585	*VDAC1*	breast	2	metaplastic carcinoma, carcinoma	2	2	1	2	2
DGCR8	ENSG00000078549	*ADCYAP1R1*	breast	1	carcinoma	1	1	1	1	1
DGCR8	ENSG00000131095	*GFAP*	breast	1	carcinoma	1	1	1	1	1
FUS	ENSG00000131095	*GFAP*	breast	1	carcinoma	1	1	1	1	1
AUH	ENSG00000244731	*C4A*	breast	2	ductal carcinoma, carcinoma	2	2	2	2	2

BCCIP	ENSG00000244731	*C4A*	breast	2	ductal carcinoma, carcinoma	2	2	2	2	2
BUD13	ENSG00000244731	*C4A*	breast	2	ductal carcinoma, carcinoma	3	3	2	2	3
CSTF2T	ENSG00000244731	*C4A*	breast	1	carcinoma	1	1	1	1	1
EIF4A3	ENSG00000244731	*C4A*	breast	2	ductal carcinoma, carcinoma	2	2	1	2	2
FAM120A	ENSG00000244731	*C4A*	breast	2	ductal carcinoma, carcinoma	2	2	2	2	2
GTF2F1	ENSG00000244731	*C4A*	breast	2	ductal carcinoma, carcinoma	2	2	2	2	2
HNRNPA1	ENSG000002	*C4A*	breast	2	ductal carcinoma, carcinoma	2	2	2	1	1

	44731									
HNRNPC	ENSG000002 44731	*C4A*	breast	1	carcinoma	1	1	1	1	1
HNRNPK	ENSG000002 44731	*C4A*	breast	2	ductal carcinoma, carcinoma	2	2	2	1	1
HNRNPUL1	ENSG000002 44731	*C4A*	breast	2	ductal carcinoma, carcinoma	2	2	2	1	1
IGF2BP1	ENSG000002 44731	*C4A*	breast	2	ductal carcinoma, carcinoma	2	2	2	2	2
IGF2BP3	ENSG000002 44731	*C4A*	breast	2	ductal carcinoma, carcinoma	2	2	2	2	2
LARP7	ENSG000002 44731	*C4A*	breast	2	ductal carcinoma, carcinoma	2	2	2	1	1

LIN28B	ENSG000002447 31	*C4A*	breast	2	ductal carcinoma, carcinoma	2	2	2	2	2
PRPF8	ENSG000002447 31	*C4A*	breast	2	ductal carcinoma, carcinoma	2	2	2	1	1
RBFOX2	ENSG000002447 31	*C4A*	breast	2	ductal carcinoma, carcinoma	2	2	2	1	1
SF3A3	ENSG000002447 31	*C4A*	breast	2	ductal carcinoma, carcinoma	2	2	2	2	2
SF3B4	ENSG000002447 31	*C4A*	breast	2	ductal carcinoma, carcinoma	2	2	2	2	2
SLTM	ENSG000002447 31	*C4A*	breast	2	ductal carcinoma, carcinoma	2	2	2	1	1
SMNDC1	ENSG000002	*C4A*	breast	2	ductal carcinoma, carcinoma	2	2	2	2	2

	4473<br>1									
SND1	ENS<br>G00<br>0002<br>4473<br>1	*C4A*	breast	2	ductal carcinoma, carcinoma	2	2	2	2	2
SRSF1	ENS<br>G00<br>0002<br>4473<br>1	*C4A*	breast	2	ductal carcinoma, carcinoma	2	2	2	2	2
SRSF7	ENS<br>G00<br>0002<br>4473<br>1	*C4A*	breast	2	ductal carcinoma, carcinoma	2	2	2	2	2
SRSF9	ENS<br>G00<br>0002<br>4473<br>1	*C4A*	breast	2	ductal carcinoma, carcinoma	2	2	2	2	2
TRA2A	ENS<br>G00<br>0002<br>4473<br>1	*C4A*	breast	2	ductal carcinoma, carcinoma	2	2	2	1	1
U2AF1	ENS<br>G00<br>0002<br>4473<br>1	*C4A*	breast	2	ductal carcinoma, carcinoma	2	2	2	2	2

Supplementary Table S6. List of RNA binding proteins interacting with the FTD-breast cancer comorbid genes

GeneID	GeneName	GeneType	RBP	ClusterNum	ClipSiteNum
ENSG00000117020	AKT3	protein_coding	TARDBP	84	783
ENSG00000213585	VDAC1	protein_coding	TARDBP	12	284
ENSG00000078549	ADCYAP1R1	protein_coding	TARDBP	9	65
ENSG00000131095	GFAP	protein_coding	MBNL2	6	41

Supplementary Table S7. RBP-mRNA Interaction for FTD-breast cancer comorbid genes

GeneID	GeneName	GeneType	PairGeneID	PairGeneName	PairGeneType	Interaction	AlignScore	MaxConMatch
ENSG00000117020	AKT3	protein_coding	NR_146151_blat	RNA45SN3	rRNA	25	26	10
ENSG00000213585	VDAC1	protein_coding	SSU-rRNA	SSU-Rrna	rRNA	119	38	12
ENSG00000078549	ADCYAP1R1	protein_coding	NR_146151_blat	RNA45SN3	rRNA	5	17.5	9
ENSG00000244731	C4A	protein_coding	NR_146151_blat	RNA45SN3	rRNA	6	19	7
ENSG00000131095	GFAP	protein_coding	NR_146151_blat	RNA45SN3	rRNA	5	17	8

Supplementary Table S8: Pathogenicity Score of for FTD-breast cancer comorbid genes measured with various pathogenicity score tools

Genes	CADD Phred Score	CADD Score	DANN Phred Score	DANN Score	regBase PAT Phred Score	regBase PAT Score	Eigen Phred Score	Eigen Score	FATHMM-MKL Phred Score	FATHMM-MKL Score	Eigen _PC Phred Score	Eigen _PC Score	ReMM Phred Score	ReMM Score	Fit Cons Phred Score	Fit Cons Score	Geno Canyon Phred Score	Geno Canyon Score	LINSIGHT Phred Score	LINSIGHT Score	CDTS Phred Score	CDTS Score
AKT3	3.356	0.0753	6.2867	0.764	1.1319	0.0136	4.6836	-0.0339	8.4019	0.2445	5.147	-0.1476	0.7211	0.013	5.6188	0.0744	2.8026	0.0045	2.3568	0.0449	5.0283	-1.865
VDAC1	27.3171	5.8886	31.0655	0.9984	25.8373	0.9653	N/A	0	33.4932	0.9954	0	N/A	0.989	22.8892	27.3382	0.7015	99.3328	1	0	N/A	13.5348	-6.9825
GFAP	13.6996	1.5755	7.1954	0.7867	4.9235	0.0658	14.7234	0.874	7.0101	0.203	15.8363	1.2607	13.6916	0.845	13.2325	0.1218	12.4471	1	13.7814	0.1907	6.5415	-3.0822
C4A	26.4255	5.6079	24.7412	0.9946	22.5452	0.9382	0	NA	21.115	0.9794	0	N/A	18.705	0.964	20.8745	0.5776	4.7749	0.7376	0	N/A	0	N/A
ADCYAP1R1	11.6226	1.1787	2.8942	0.6248	6.245	0.0969	13.0173	0.6259	7.8497	0.226	7.467	-0.0809	11.9722	0.795	1.8046	0.0537	5.9097	0.9836	14.1871	0.2167	2.2688	1.6776

Key/Legend:

Phred Score = confidence integral of pathogenicity

Score = Raw Score

Likley Pathogenic

Supplementary Table S9. List of 3'aQTL variants from Brain Frontal Cortex tissue for *AKT3* and *GFAP* genes.

	AKT3	
**rs ID**	**Variant ID**	**3'aQTL P-value**
rs2502342	chr1_242907165_C_T_b38	0.00003769
rs2998662	chr1_242903675_G_C_b38	0.00001787
rs2998661	chr1_242903316_G_A_b38	0.00001246
rs12077950	chr1_242902067_T_C_b38	0.00003583
rs9725721	chr1_242901771_G_A_b38	0.00003583
rs6429391	chr1_242901126_T_G_b38	0.00003583
rs6429389	chr1_242900607_C_T_b38	0.00003583
rs12075066	chr1_242900095_T_C_b38	0.00003583
rs12078540	chr1_242899843_G_A_b38	0.00003583
rs12073551	chr1_242898757_T_A_b38	0.00003583
rs12087532	chr1_242898633_C_A_b38	0.00003583
	GFAP	
rs12941832	chr17_44910114_G_T_b38	0.00007407
rs34902223	chr17_44870901_C_T_b38	0.00007407
rs9895349	chr17_44866621_C_T_b38	0.00007407
rs4793148	chr17_44762183_C_A_b38	0.0000448
rs3764840	chr17_44760112_C_T_b38	0.0000448
rs3760382	chr17_44758548_T_C_b38	0.0000448
rs4426386	chr17_44758127_A_G_b38	0.0000448
rs2337848	chr17_44754386_G_A_b38	0.0000448
rs9893320	chr17_44747280_T_C_b38	0.0000448
rs9911454	chr17_44743640_C_T_b38	0.0000448
